# Application of anatomy-based spacing of electrode contacts for achieving a uniform semitonal resolution: A novel concept in cochlear implant electrode design

**DOI:** 10.1038/s41598-024-53070-8

**Published:** 2024-02-01

**Authors:** Isra Ali Aljazeeri, Abdulrahman Hagr

**Affiliations:** 1 King Abdullah Ear Specialist Center (KAESC), College of Medicine, King Saud University Medical City (KSUMC), King Saud University, PO Box 245, 11411 Riyadh, Saudi Arabia; 2 Otolaryngology and Ophthalmology Specialized Aljaber Hospital, Ministry of Health, Ahsa, Saudi Arabia

**Keywords:** Outcomes research, Preclinical research

## Abstract

Using anatomy-based fitting, we can determine the place-specific map with individualized center frequencies for each electrode contact that is a closer match to the natural pitch-place of the cochlea. The primary objective of this study is to evaluate the tonal presentation across the electrode array and to calculate the semitone difference between each adjacent pair of contacts according to their anatomy-based map. The secondary objective is to determine the distancing of the contacts that would result in an equal semitone difference with a uniform tonal presentation. A total of 167 ears were included in this retrospective study. The frequencies across the electrode arrays were found to be unequally presented. The semitonal condensations were higher in the apical inter-contact spaces compared to the basal inter-contact spaces, being 3.0–2.3 semitones/mm (Kruskal Wallis test, *p* < 0.000). The anatomy-based spacing of the electrode contacts was larger in the basal inter-contact spaces compared to the apical inter-contact spaces, ranging from 1.92 to 1.48 mm. In conclusion, the current electrode designs do not have uniform tonal representation throughout the electrode array. There is a more condensed tonal presentation in the apical electrodes than in the basal electrodes, resulting in a lower tonal resolution in the apical region.

## Introduction

Cochlear implantation is the mainstay treatment for patients with sensorineural hearing loss who do not benefit from hearing aids^1^. Since the first cochlear implantation surgery, there has been a continuous effort to improve the design and function of the device^2,3^, and the satisfaction of the cochlear implant (CI) patients^4^.

CIs are designed to replace the normal cochlea, particularly with hearing preservation arrays—they bypass the damaged or dysfunctional areas^1,5^. Therefore, to better understand and improve the designs of the CI, there is a need to understand how a normal cochlea function. The normal cochlea has a tonotopic representation of the frequencies, making each part most sensitive to a specific frequency, which is called its characteristic frequency^6^. The tonotopicity of the cochlea makes it more sensitive to higher frequencies in the basal region and lower frequencies in the apical region^7^.

The programming of CIs includes assigning specific frequency range to each electrode contact. This programming map can either be assigned using a default frequency map or, in the recent practice, to be personalized according to the patient’s cochlear anatomy^8^. The latter is known as anatomy-based frequency mapping^9^.

In default mapping, the frequencies assigned to each electrode contact follow a frequency table with fixed frequencies for all patients. Although these frequencies were based on the Greenwood equation to follow the tonotopicity of the cochlea, this default map is not personalized. Recent studies have argued that using default frequency mapping for speech processor programming results in a mismatch between the stimulating frequency presented by some of the electrode contacts and the characteristic frequency that corresponds to the real place of those contacts in the cochlea^10^. There are three reasons for the occurrence and degree of mismatch. First, there are diverse ranges of cochlear duct lengths^11,12^. Second, electrode lengths differ from one array type to the other^13^. Third, electrode contacts can be positioned in a different site of the cochlea depending to the degree of surgical insertion^14,15^.

To date, all electrode arrays have constant, and algebraically equal, spacing between their contacts. This method of spacing would results in a non-homogenous presentation of frequencies along the electrode array, in terms of the number of semitones. We assume that similar semitone differences would result in more uniform hearing representation. However, due to the logarithmic nature of the frequency differences, the electrode spacing should not be algebraically equally divided. To enable similar tonal resolution, electrode arrays need to have a smaller electrode spacing in the apical region compared to the basal region.

The primary objective of this study is to evaluate the condensation of the tonal presentation across the electrode array and to calculate the semitone differences between adjacent pairs of electrodes according to their anatomy-based frequency map. The secondary objective is to determine the spacing of the electrode contacts that results in an equal semitone difference with uniform tonal presentation.

## Methodology

The medical data of patients in the CI registry who underwent cochlear implantation at a tertiary referral center for neuro-otology and CI surgery were retrospectively reviewed. Before conducting this study, the approval of the Institutional Review Board of King Saud University Hospital was obtained (Ref. No. 20/0091/IRB, Project No. E-20-5387).

### Patient selection

The preoperative computed tomography (CT) scans of 167 ears of 100 patients who underwent cochlear implantation at King Abdullah Ear Specialist Center were reviewed. No limitation was set for the chosen subjects regarding their age, sex, or number of implantations. All implanted electrodes that were compatible with the software used in this study were included. Patients with inner ear anomalies, cochlear ossification, cochlear otosclerosis, an otic capsule involving temporal bone fractures, or any bone diseases involving the temporal bone were excluded.

### Imaging

Images were obtained using a 512-slice multidetector-row CT scanner (General Electric Healthcare, Milwaukee, WI). The following scanning parameters were used: axial plane, 0.625 mm slice thickness, 230 mAs, 140 kV, rotation time 1 s, with 0.3 mm reconstruction in the axial and coronal views.

### Anatomy-based prediction of frequency allocation for each electrode

The preoperative CT images were uploaded to the Otoplan V.03 DICOM viewer and surgical planning software (CAScination AG, Bern, Switzerland). This software predicts the personalized, anatomy-based frequency allocation of each electrode according to the imaging-based cochlear duct length (CDL) and the length of the electrode array.

Semitonal differences were defined as the differences between the anatomy-based frequency of two adjacent electrode contacts and were measured in semitones.

The inter-contact spacing refers to the physical distance between the two adjacent electrode contacts.

**Figure 1:**
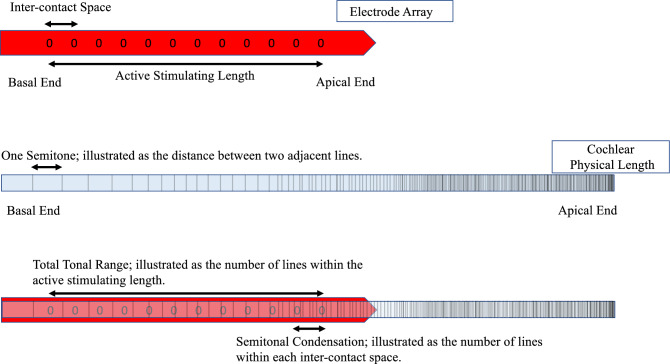
Illustrates the concepts used in this article.

Semitone condensation was calculated by dividing the semitone differences between adjacent electrode contacts by their spacing distance. Tonal resolution was defined as 1 over semitone condensation (Fig. 1).

Anatomy-based electrode spacing was determined by dividing the frequency range of the anatomy-based array frequency coverage into equal semitonal distances. After determining the frequencies that would result in equal semitonal distances, the corrected Greenwood equation was used to determine the spacing of each pair of adjacent electrodes^16–19^.

In this study all the included electrode types were from the same manufacturer, Med El (Innsbruck, Austria), that is compatible with the Otoplan software and has twelve contacts in each electrode array type.

The total lengths of the electrode arrays were 24, 28, and 31 mm for the Form-24, Flex-28, and Standard types of electrode arrays, respectively. These electrodes have an active stimulating range of 18.7, 23.1 and 26.4 mm for the Form-24, Flex-28, and Standard types of electrode arrays, respectively. The inter-contact spacing of these electrode arrays is 1.7 mm, 2.1 mm, and 2.4 mm for the Form-24, Flex-28, and Standard types of electrode arrays, respectively.

In Med El devices, discussed in this article, the most apical electrode is referred to as the first while the most basal one is the twelfth one.

### Data collection and analysis

Data collection was performed using the Excel software (version 16.3; Microsoft, Seattle, WA). The statistical analyses were performed using IBM SPSS software (version 23.0; IBM Corp., Armonk, NY). Statistical significance was set at *p* < 0.05. The inter-rater reliability was evaluated using Cronbach’s alpha. The means of the measurements taken by the two reviewers were used for further analyses.

## Results

A total of 167 implanted ears in 100 patients (60 males, 40 females) were included. Overall, 105 (62.8%) ears received the Form-24 electrode array, 53 (31.7%) received the Flex-28, and nine (5.3%) received the Standard electrodes.

It was found that the two reviewers' electrode frequency readings were homogenous and had a high level of internal consistency (Cronbach's alpha = 0.96).

The semitone differences between the two most apical electrode contacts were the lowest in the Form-24 electrode array type. The semitone differences in the Form-24 electrodes ranged from 4.9 semitones in the most apical contacts to 4.1 semitones in the last two basal electrodes, 6.7–4.8 semitones in the Flex-28, and 8.9–5.4 semitones in the Standard electrode array types. The semitone differences were significantly higher in the apical spaces and decreased towards the most basal inter-contact spaces (Kruskal Wallis test, Chi-Square value from 108 to 111, *p* < 0.000) (Supplementary File 1, Fig. 2).

**Figure 2:**
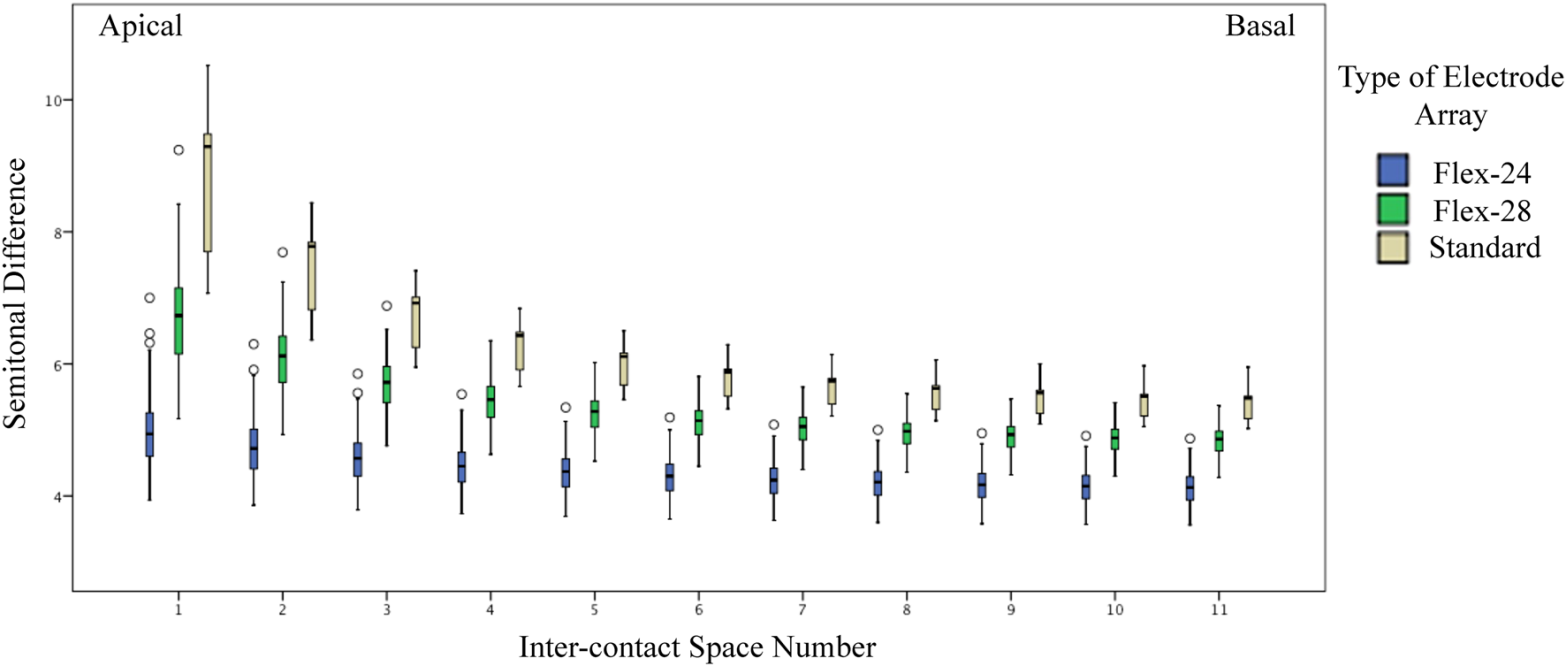
The anatomy-based semitonal differences between adjacent electrode contacts in the three types of electrode arrays.

**Figure 3:**
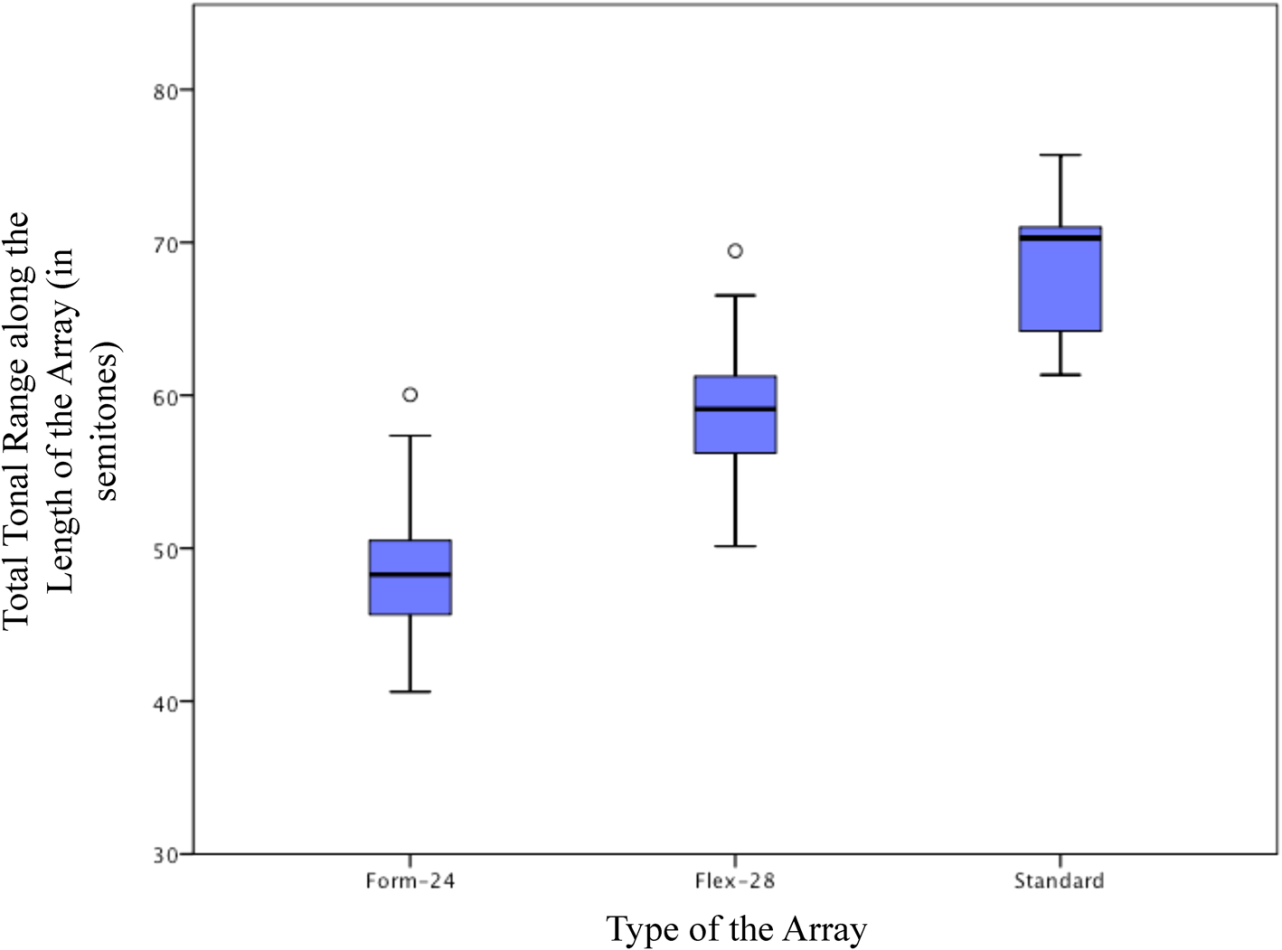
The tonal range of the anatomy-based frequencies for each electrode array type.

The longest electrode array type, the standard electrode, demonstrated the highest range of tonal representation followed by the shorter Flex-28, and Form-24 (Fig. 3).

**Figure 4:**
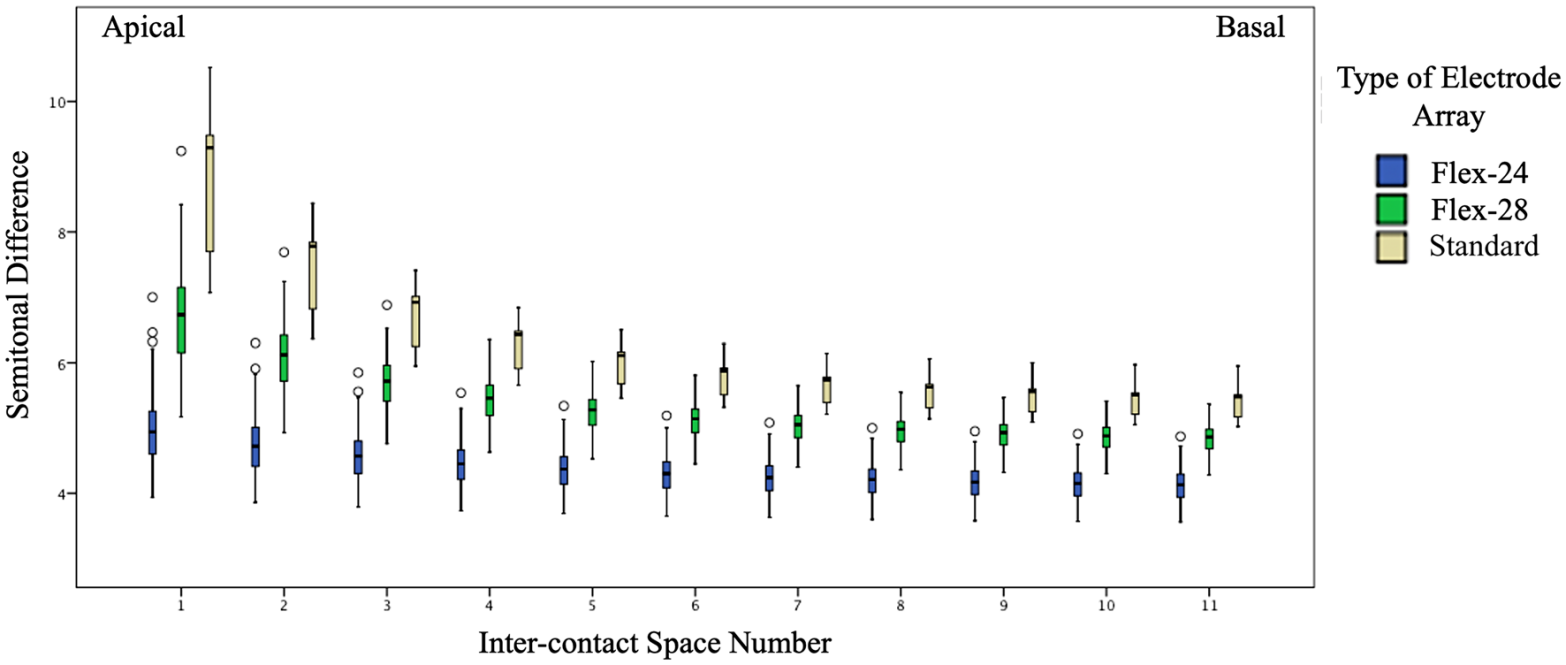
The anatomy-based semitonal condensation of the presented stimulations in the electrode contact spaces for each type of the electrode array.

The semitonal condensation was higher in the apical electrodes and decreased towards the basal electrode contact spaces, starting from 3.05 semitone/mm at the first apical space, and decreasing to 2.3 semitone/mm at the last basal space. The semitonal condensation of the inter-contact spaces along the array was found to be significantly different (Kruskal Wallis test, Chi-Square value 823.1, *p* < 0.000). The longer, Standard, electrode array type demonstrated the highest semitonal condensation followed by the Flex-28 and lastly Form-24 in the apical region. This relation was reversed in the basal region, with the Standard electrode demonstrating the lowest semitonal condensation (2.25 semitone/mm) compared to the Flex-28 (2.3 semitone/mm) and the highest condensation was seen in Form-24 (2.4 semitone/mm). The difference in the semitonal condensation between the three electrode array types was found to be significant (Kruskal Wallis test, Chi-Square value 33.1, *p* < 0.000) (Fig. 4).

**Figure 5:**
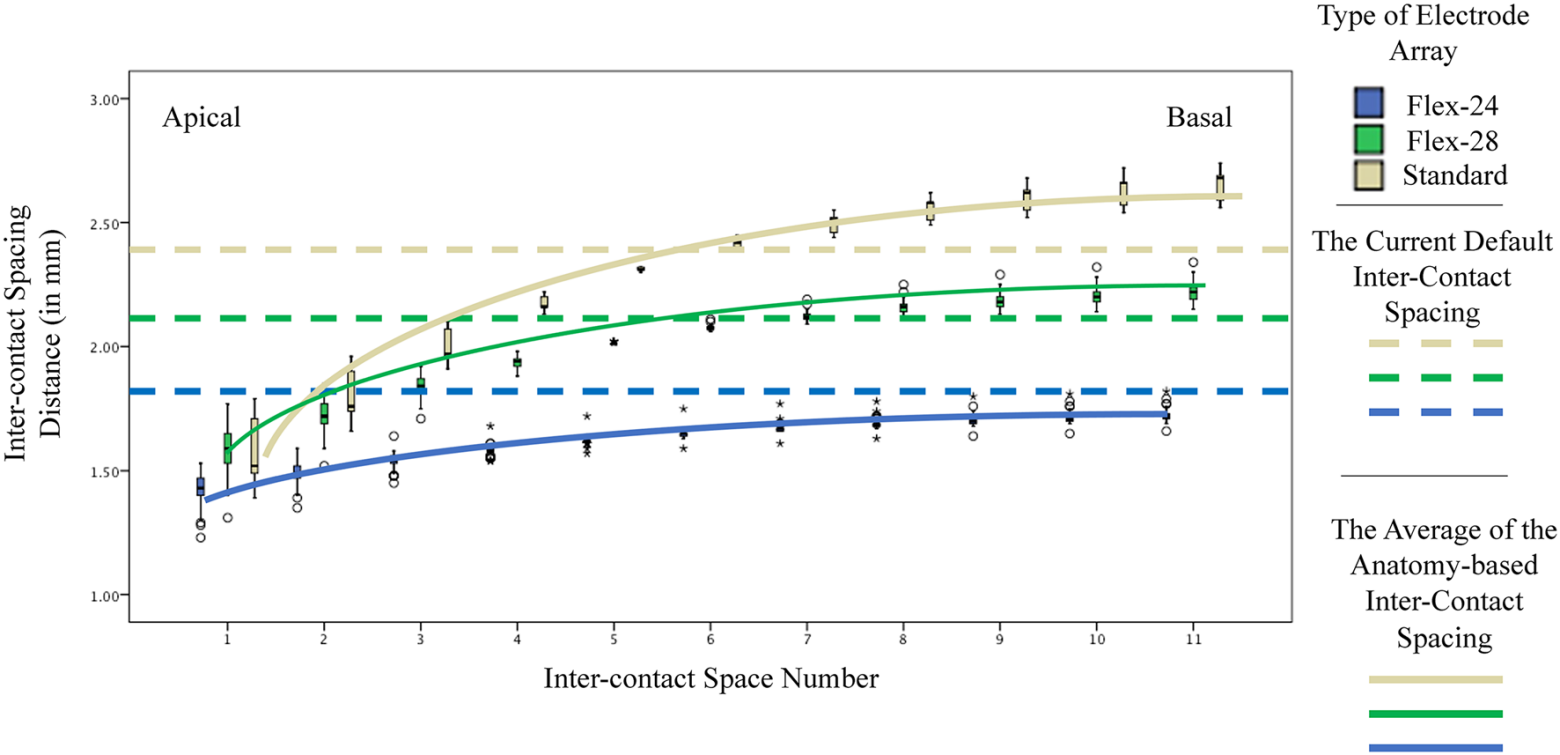
The anatomy-based inter-contact electrode spacings shown in boxplot. The error bar stands for the SD, the outliers shown in white circles, while the extreme scores are illustrated as a star. The anatomy-based spacings were higher than the actual spacings in the basal electrodes and lower than the actual spacing in the apical region. The dashed lines denote the actual spacing in the current electrode array designs.

The anatomy-based inter-contact spacing revealed that for an equal inter-contact semitonal condensation there is a need for a 1.48 mm inter-contact space in the most apical region and this distance needs to increase to 1.92 mm in the most basal region of the electrode array. The anatomy-based inter-contact spacing were significantly different across the electrode arrays (Kruskal Wallis test, Chi-Square value from 88 to 119, *p* < 0.000) (Table 1, Fig. 5).

**Table 1:** The anatomy-based estimated electrode contact spacing between the adjacent electrode contacts between the three types of electrode arrays.

Type of Electrode Array		1st Space	2nd Space	3rd Space	4th Space	5th Space	6th Space	7th Space	8th Space	9th Space	10th Space	11th Space
The anatomy-based electrode contact spacing												
Form 24	Mean	1.42	1.48	1.54	1.58	1.61	1.64	1.66	1.68	1.70	1.71	1.72
	SD	0.05	0.03	0.02	0.01	0.01	0.01	0.01	0.01	0.01	0.01	0.02
Flex 28	Mean	1.58	1.72	1.84	1.93	2.01	2.07	2.12	2.15	2.18	2.20	2.21
	SD	0.08	0.06	0.03	0.01	0.00	0.01	0.01	0.02	0.03	0.03	0.03
Standard	Mean	1.57	1.79	2.00	2.17	2.31	2.41	2.49	2.55	2.60	2.63	2.65
	SD	0.14	0.10	0.06	0.03	0.00	0.021	0.03	0.04	0.05	0.06	0.06

The first space refers to the tonal difference between the first and the second electrode contacts in the most apical region, while the 11th space is the tonal difference between the 11th and 12th electrode contacts.

Two-way ANOVA showed that both the type of electrode array and the inter-contact space number are significantly affecting the estimated spacing for equal semitonal differences. (F value 36,277 & 2738, partial Eta squared showing an effect size of 0.97 & 0.93 respectively and *p* < 0.001 for both) The interaction of the two factors also showed to be significantly affecting the estimated spacing. (F = 9.04, partial Eta squared showing an effect size of 0.80 with *p* < 0.001) It must be mentioned that the Levene’s test shows that the variances are not homogenous. (F value of 35.86, *p* < 0.001).

## Discussion

This study demonstrated the variability in the tonal representation across the electrode, using anatomy-based frequency mapping. Apical electrode spaces demonstrated greater tonal condensation with higher ranges of semitone differences between adjacent electrode contacts compared to basal electrode spaces.

Significant improvements in the understanding of cochlear anatomy have been achieved following the development of the synchrotron radiation phase-contrast imaging technique^7^. Using the findings of previous studies, corrections have been made to the equations by which anatomy-based spacings are determined^7^. In this study, OC map was used to determine the anatomy-based frequency allocations. It must be mentioned that uncertainty still exists on where along the signal pathway, the cochlear stimulation happens. For the lateral wall electrode arrays that were included in this study, the OC is assumed to be the first site of stimulation by the electrical signal due to the more proximity of the OC to the laterally placed array^20^.

Previous studies have found that the frequency-to-place mismatch differs across the electrode arrays and is greater in apical electrode contacts^21^. Having perfect frequency-to-place matching would result in worse tonal resolution in the apical region, as demonstrated by the current results. Of the included electrode designs, the highest anatomy-based frequency resolution in the basal spaces was found in the standard electrodes, and the lowest anatomy-based frequency resolution in the apical spaces was also found in the standard electrodes. In the apical region, semitonal condensation was highest in the standard electrodes, followed by the Flex-28 and Form-24 arrays; however, this order was reversed in the basal electrode region. This phenomenon can be explained by the deeper insertion of the Standard electrode causing a lower frequency representation in a more sensitive apical regions of the cochlea that has a condensed tonal representation. While in the more basal area the condensation is more affected by the electrode spacing which is higher in the Standard electrode causing a lower condensation.

**Figure 6:**
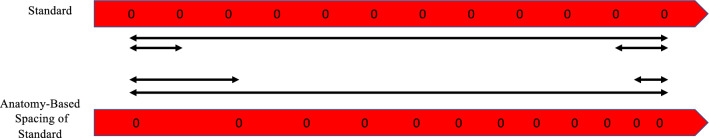
Showing an illustration of the default and anatomy-based spacing in the Standard array. In comparison to the default spacing, the basal contacts are farther apart in the anatomy-based spacing, and the apical contacts are closer together. *Note*: the distances in this figure are not calculated and are for illustration purpose only.

The anatomy-based inter-contact spacing findings demonstrated that more space is needed in the basal electrode contacts compared to the apical spaces to achieve equal and uniform inter-contact semitonal resolution across the electrode array (Fig. 6). One key consideration is that the magnitude of anatomy-based electrode spacing is dependent on personalized cochlear duct length. Interestingly, there was small variability in the anatomy-based spacing within each CI electrode array type. This creates the opportunity to develop a new electrode design with constant inter-contact spacing for use across a wide range of cochlear duct lengths. It must be kept in mind however that although these variabilities are small on millimetric spacing scale, they are actually not that small, when you transfer them back onto a semitonal distances. Which means that although using this new spacing would improve the tonal representation, but it still would lack a perfect uniform tonal spacing due to the individual differences in the CDL.

As the anatomy-based frequency allocations of the electrode contacts were used to calculate the inter-contact spacing, the total active stimulating length of the electrode with the anatomy-based spacing was assumed to be constant.

**Figure 7:**
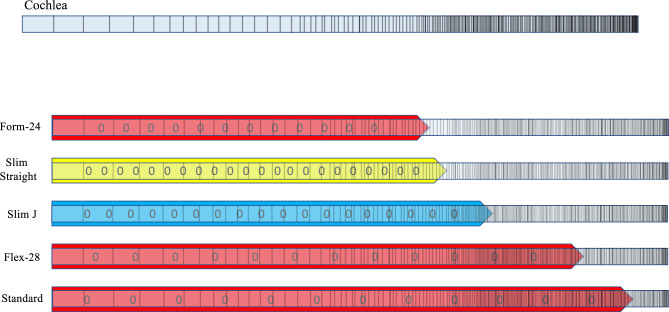
Showing an illustrative figure of the default inter-contact spacing for lateral array electrodes of different manufacturers.

Although we included only one manufacturer’s electrode arrays in this study, the spacing method suggested in this study can be applied to the other manufacturers’ arrays as well. The calculations must consider the number of the contacts and the active stimulating length of the array. Currently there is no electrode array design that has an anatomy-based spacing of the contacts and all contacts are spaced equally in their physical distance rather than the tonal distances of the cochlea. Which means all the arrays used have an ununiform tonal representation (Fig. 7).

A limitation of this study is that the clinical significance of anatomy-based spacing needs to be further studied after electrode spacing is changed. It is also possible to study this spacing method using virtual electrode contact spacing without the need to change the electrode design^22,23^. Furthermore, the anatomy-based spacing were measured on the assumption of full surgical insertion, which is not always possible. The final configuration of the electrode inside the cochlea can also affected by the spatial geometry of the cochlea resulting in different localization of the contact than expected by preoperative imaging. Even with these limitations, generally concluding, the more apical electrode contacts would still need to be closer to each other compared to the basal electrode contacts to enable a more uniform frequency presentation.

Other factors that affect the location of the stimulation are the spread of excitation in monopolar stimulation mode, which can lead to off-site and cross-turn stimulation in areas which can limit any approach aiming to improve the frequency to place matching.

A number of strategies have aimed to solve the spread of excitation effect, including continuous interleaved sampling (CIS), n-of-m and advanced combination encoder, and precompression for spread of excitation strategies^24–27^.

Another matter of importance is the fact that an apically closer spacing of electrode contacts might result in signal interaction due to the spread of the excitation^28^. This can limit the benefit of these contacts. However, the spread of excitation is affected by numerous variables including the programming parameters that can be calibrated in a way to preserve contact functioning without signal interaction^29,30^.

## Conclusion

Uniform algebraic electrode contacts spacing results in an unequal tonal resolution across electrode arrays, with better resolution in the basal compared to the apical inter-electrode spaces. The anatomy-based electrode contact spacing would require wider spacing in the basal electrode contacts and narrower spacing in the apical electrode contacts. The small variability of the anatomy-based contact spacing despite the variability in patient cochlear duct lengths would enable the potential development of a constant electrode contact spacing that would fit most patients.

### Supplementary Information

Supplementary Information.

## Data Availability

The data used in this study are available upon requests send to the corresponding author’s email: dr.isra.aljazeeri@gmail.com.
