# Supplementary material for: Application of anatomy-based spacing of electrode contacts for achieving a uniform semitonal resolution: A novel concept in cochlear implant electrode design
Source: Sci Rep. 2024 Feb 1;14:2645. doi: 10.1038/s41598-024-53070-8 (PMC10834526; doi:10.1038/s41598-024-53070-8)
Supplement: Supplementary file 1 — Supplementary Information. [file 41598_2024_53070_MOESM1_ESM.docx]

|  | Type of electrode array |  | 1^st^ space | 2^nd^ space | ^3rd^ space | 4th space | 5th space | 6th space | 7th space | 8th space | 9th space | 10th space | 11th space |
| --- | --- | --- | --- | --- | --- | --- | --- | --- | --- | --- | --- | --- | --- |
| Tonal differences between the adjacent electrode contacts in semitones. | Form 24 | Mean | 4.97 | 4.74 | 4.57 | 4.45 | 4.36 | 4.29 | 4.24 | 4.20 | 4.17 | 4.14 | 4.12 |
|  |  | SD | .55 | .45 | .38 | .34 | .31 | .29 | .28 | .27 | .26 | .26 | .25 |
|  | Flex 28 | Mean | 6.70 | 6.09 | 5.69 | 5.43 | 5.24 | 5.11 | 5.02 | 4.95 | 4.90 | 4.86 | 4.83 |
|  |  | SD | .74 | .52 | .40 | .33 | .28 | .26 | .24 | .22 | .22 | .21 | .21 |
|  | Standard | Mean | 8.92 | 7.51 | 6.75 | 6.29 | 6.00 | 5.80 | 5.66 | 5.56 | 5.50 | 5.45 | 5.42 |
|  |  | SD | 1.25 | .75 | .53 | .42 | .36 | .32 | .30 | .29 | .29 | .28 | .29 |
|  | Total | Mean | 5.73 | 5.32 | 5.04 | 4.86 | 4.73 | 4.63 | 4.56 | 4.51 | 4.47 | 4.44 | 4.42 |
|  |  | SD | 1.28 | .95 | .76 | .66 | .59 | .54 | .51 | .49 | .48 | .47 | .47 |

Supplementary 1. The anatomy-based semitonal differences between the adjacent electrode contacts between the three types of electrode arrays. The first space refers to the tonal difference between the first and the second electrode contacts in the most apical region, while the 11^th^ space is the tonal difference between the 11^th^ and 12^th^ electrode contacts.
